# Building engagement to support adoption of community-based substance use prevention initiatives

**DOI:** 10.1186/s12889-022-14496-9

**Published:** 2022-11-29

**Authors:** Tanya Halsall, Kianna Mahmoud, Annie Pouliot, Srividya N. Iyer

**Affiliations:** 1 University of Ottawa Institute of Mental Health Research at The Royal, 1145 Carling Avenue, Ottawa, ON K1Z 7K4 Canada; 2 Department of Neuroscience, 1125 Colonel By Drive, Ottawa, ON K1S 5B6 Canada; 3 Faculty of Medicine, Dalhousie University, 5849 University Ave, Halifax, NS B3H 4R2 Canada; 4 Public Health Agency of Canada, 130 Colonnade Rd, A.L. 6501H, Ottawa, ON K1A 0K9 Canada; 5 ACCESS Open Minds (pan-Canadian youth mental health research network), Montreal, Quebec Canada; 6 Douglas Mental Health University Institute, Montreal, Quebec Canada; 7 Department of Psychiatry, McGill University, 1033 Av. des Pins, Montreal, QC H3A 1A1 Canada

**Keywords:** Icelandic prevention model, Community-based health promotion, Implementation science, Qualitative methods, Public health, Collaboration, Complexity science, Evaluation

## Abstract

**Background:**

System-level approaches that target social determinants of health are promising strategies to support substance use prevention, holistic youth development and wellbeing. Yet, the youth services system is largely based on individual-focused programs that do not adequately account for social determinants of health and place the responsibility for wellness on the individual. There is a need to understand how to enhance adoption of complex system-level approaches that support comprehensive youth development. The Icelandic Prevention Model (IPM) represents a collaborative initiative that takes an ecological, system-level approach to prevent substance use and promote wellness in youth. This research was designed to examine key stakeholder perceptions to better understand social motivations and contextual complexities that influence stakeholder support to garner community-level adoption of the IPM in a rural Canadian community.

**Methods:**

This research applies a case study approach using qualitative interviews to explore strategies to support uptake in the early stages of IPM adoption associated with developing community buy-in and acceptance. A thematic analysis was applied using QSR NVivo.

**Results:**

Nine interviews were conducted with community partners leading the implementation of the IPM. Three over-arching themes emerged from the data: 1) Motivating influences 2) Strategies to develop buy-in, and 3) Resistance to the adoption of the IPM. Findings reflect issues that affect behaviour change in system transformation in general as well as upstream prevention and the IPM, in particular.

**Conclusions:**

The findings from this research describe critical insight derived from implementing community-driven initiatives that are designed to support health promotion. It contributes new scientific knowledge related to implementation of complex system-level innovations and practical information that is useful for communities interested in implementing the IPM or following similar approaches to prevent substance use.

## Background

Researchers have acknowledged that individual-focused programs and services, by themselves, are not a viable way to reduce population health inequities [1], support substance use prevention [2] and promote comprehensive youth development [3, 4]. Further, efforts must include a focus on system-level approaches that target social determinants and environmental factors that influence overall health and wellbeing [5, 6]. Yet, there continues to be an over-reliance on individual-focused programs that focus on intentional behaviour change that are ineffective for creating population-level health outcomes [1, 7–10]. Often this is the result of lifestyle drift, a process whereby initiatives start “with a commitment to dealing with the wider social determinants of health but end up instigating narrow lifestyle interventions on individual behaviours’ ([1, 10, 11]; p. 323). It is critical to enhance our understanding of how to support adoption of these complex system-level approaches [12–16]. Further, it is important to better understand the mechanisms that influence a shift in focus toward individual programming in order to enhance population-level interventions in the future.

Multi-level ecological approaches require system-level partnerships to be successful, therefore adoption of these efforts necessitate the consideration of complex system issues that take context and stakeholder perspectives into account. When considering the uptake and spread of these innovations, it is useful to apply complexity science [13, 17], systems thinking [14] and social science frameworks to better understand underlying mechanisms, adaptations and social influences that affect uptake and implementation [13]. The Icelandic Prevention Model (IPM) represents a collaborative model that takes an ecological, system-level approach to prevent substance use in youth [18, 19]. This paper applies a case study method to examine the early stages of community engagement in adopting the IPM in a rural Canadian community. We examine key stakeholder perceptions to better understand social motivations and contextual complexities that influence behaviour change to garner community-level adoption of the model.

### Community-based health promotion and substance use prevention

Community-based health promotion programs have been defined as initiatives that integrate the following criteria: they apply ecological approaches, are tailored to community needs and engage community members within participatory strategies [20]. Ecological approaches derive from the bioecological model, a theory designed to understand human development [6]. This model views the developing individual within a dynamic context and accounts for the mutual interactions between the individual and developmental environment. This perspective is based on the assumptions that developmental systems must be viewed as holistic, dynamic, nonlinear and complex and that within these systems, developing individuals possess individual agency and influence over their surroundings [21]. Recent frameworks that take a holistic approach to the measurement of well-being such as the Canadian Index of Child and Youth Wellbeing [22] and the Public Health Agency of Canada’s Positive Mental Health Surveillance Indicator Framework [23] help to illustrate the systemic nature of development and the need to consider a range of health determinants within program and policy design.

Multi-level approaches take account of a range of influences across developmental contexts, such as interventions that include family, school and policy-focused components, thus strengthening their potential for influence on individual development (see [3, 18, 19, 24, 25]. “A complex systems model of public health conceptualises poor health and health inequalities as outcomes of a multitude of interdependent elements within a connected whole” ([1] p. 2602). Therefore, system-level strategies involve collaboration from intersectoral partners from across the system who are implicated in supporting youth development through a range of influential pathways [1, 3, 10, 26, 27].

Youth substance use and the associated harms are a population health issue. In Canada, 44% of secondary students have used alcohol over the past 12 months and 18% have used cannabis [28]. Some factors that have been found to influence youth substance use behaviours include social norms [29, 30], personal disposition [31], processes of brain development [8, 32] and time use patterns [33, 34]. Similar to other population health issues, individual-focused substance use prevention approaches that use education to support abstinence have not been effective [2, 35].

### Icelandic prevention model (IPM)

Although there is increasing recognition that programmatic interventions that focus on one issue will not be as effective as approaches that take a multi-level approach, there continues to be few models that actualize an ecological strategy. One exception is the IPM that has been developed and implemented on a national scale in Iceland. The IPM applies a community-based health promotion approach that places an ecological focus on developmental contexts to tailor system-level strategies to prevent substance use in youth.

Specifically, the model uses youth population surveys to examine risk and protective factors within the family, peer, school and community contexts and results are shared with community leaders to support planning of a tailored intervention [19]. Based on the first guiding principle, the model applies a “primary prevention approach that is designed to enhance the social environment” ([18], p. 4). Therefore, initiatives target their strategies to environmental factors and underlying causes, taking a more comprehensive and multi-level approach. In practice, initiatives often integrate multiple intervention functions, such as environmental restructuring and restriction (see [36]) that are tailored to population-level findings. Restriction involves the development of rules and regulations to reduce the opportunity for individuals to engage in a particular behaviour. This combination of multiple complimentary behaviour change strategies has been applied with great success in tobacco control efforts [37]. Since the model includes the collection of data that is relevant to key stakeholders and rapid dissemination of findings to community partners with a vested interest in the proposed outcomes, it aligns with the principles of utilization-focused evaluation [38]. This is illustrated in the persuasive value of the data-driven approach “… once we show the data and people see their kids they immediately ask what can we do?” ([39], p.23).

Data collection and dissemination is repeated on an ongoing basis and facilitates continued adaptations of the intervention and sustainability of the initiative. The model has been implemented in Iceland for over 20 years and trend analyses have identified a national decline in youth substance use, including a 46% reduction in 30-day youth alcohol intoxication rates before survey collection [40]. In addition, within a quasi-experimental study comparing communities that received the IPM with control communities [41], researchers found an increase in parental monitoring and youth sport participation and corresponding decreases in unstructured activities, unsupervised social gatherings and intoxication rates in communities implementing the IPM.

There are key factors to consider when supporting the adoption of the IPM within a new context. For example, there is a need to have a strong understanding of local context and culture in order to make practical adaptations [39]. Further, there are often initial challenges in building local stakeholder acceptance of the model [39]. Finally, Kristjansson and colleagues [42] suggest that there may be barriers specific to rural contexts that interfere with implementation of the IPM, such as limited resources and difficulties for community leaders to recognize the connection between health outcomes and policy.

### Implementation of innovations within complex systems

Efforts that take a system-level approach, such as the IPM, require the engagement of a range of multidisciplinary collaborators and an investment in partnership development [43, 44]. In the context of these collaborative system-level approaches, rather than maintaining a rigid focus on fidelity, implementation efforts must include considerations to ensure effective adaptation ([13, 17, 45]). Particularly within initiatives supporting scale up and spread of innovations, it is critical to examine the perspectives and sense making of local system members to better understand the underlying motivations of behaviour change [15, 16] as well as contextual dynamics and interconnectivity among actors [16]. “Indeed, working with bottom-up local stakeholders is paramount to adapting an intervention to their practices, facilitating ways to get them onboard with the intervention, in piloting it, in reflecting on progress amongst stakeholders, and in providing feedback to participants to help them embrace implementation iteratively over time” ([13], p. 8). There continues to be a need for research that examines the interactions among context, complexity, and process within translation of innovations [12] and to better understand how local stakeholders influence implementation of innovations [15].

Within these circumstances, researchers have recommended the use of qualitative methods to capture and utilize emergent findings to enhance the approach [14] and to better understand processes of development and underlying mechanisms to inform adaptations [13]. The need to explore the adoption of innovations has also been identified in the child and youth services systems [46] and in particular with respect to better understanding contextual factors and perceived acceptability of the IPM [39]. Recognizing the challenges related with lifestyle drift in population health promotion, it is important to apply knowledge developed within implementation science to support practice change, acceptance and implementation of strategies that are known to be effective.

### Purpose

This study addresses the research gaps identified above by examining contextual issues and stakeholder perspectives that influence the adoption of the IPM. We apply a case study approach, including participant observation and qualitative interviews, to explore strategies to support uptake in the early stages of community engagement. This paper describes findings derived from semi-structured interviews with key stakeholders [47].

## Method

This research is part of a larger evaluation that applies mixed methods to examine the adaptation, implementation and impact of the IPM within a rural community in Canada (see [47]. The study is guided by a pragmatic research paradigm (see [48]. Pragmatist researchers allow method choices to be driven by contextual requirements [49] and avoid a top-down approach to create space for multiple voices and perspectives within collaborative approaches [48]. Case study methods involve the in-depth exploration of a delineated entity [50, 51]. Further, case studies can be helpful when examining complex interventions and can be used to describe the in-depth processes of interventions as well as contextual features to derive explanations for how they function [52]. This is particularly useful with complex, system-level initiatives [53]. Lanark County was selected as a case study to support the in-depth examination of the implementation of the IPM within a rural Canadian community in order to inform the uptake and scaling of the IPM across Canada.

### PYLC steering committee

This research is focused on examining the implementation of the IPM within Lanark County, Ontario as Planet Youth Lanark County (PYLC). Lanark County is located in southeastern Ontario and is made up of nine municipalities that include a combination of rural and several more densely populated communities. PYLC is led by the PYLC Steering Committee, a governing body made up of local partners with significant interest in youth health promotion. Meetings take place on a monthly basis to support community engagement, identification of funding, and data collection planning and dissemination (see 19). In contrast with the typical IPM approach that is led by adult community partners, PYLC is partnering with local youth to engage them in decision-making within the initiative. This study was approved by the Royal Ottawa Health Care Group Research Ethics Board and informed consent was received from all participants.

One of the guiding principles of the IPM relates to the integration of researchers, policy makers, practitioners, and community members as collaborators [18]. TH has been participating on the PYLC Steering Committee and supporting implementation through a research and evaluation lens and initially became connected through the her involvement in the development of an evaluation guide for the Public Health Agency of Canada [54].

### Semi-structured interviews

PYLC Steering Committee meetings are open to any community members who wish to attend, however, there are about 12 members who participate on a regular basis. All PYLC Steering Committee members were invited to participate. Semi-structured interviews were conducted with nine core members over the fall of 2020 and winter of 2021 (five men and four women). All participants were community members from Lanark County and many of them represented partnering organizations. All but one participant were active members of the Steering Committee at the time of the interview. Time of involvement with the Steering Committee ranged from the period when the initiative was being established (in early 2017) to the most recent member becoming involved in late 2019. All interviews were completed by the lead author over the phone, and one was conducted online through Zoom. On average, interviews lasted about 1 hour (range: 39–95 minutes). Interview guide questions focused on issues related with context, implementation and early outcomes to capture key stakeholder perspectives of lessons learned and strategies to support acceptance and adoption of the IPM (see 46 for the interview guide). A sub-set of questions were adapted from key questions identified in the Quality Implementation Framework that are designed to facilitate adoption of innovations [55] to explore how components of the IPM are implemented (see both [18, 19].

### Data analysis

A thematic analysis [56, 57] was applied using QSR NVivo. Audio-recordings were transcribed by hand or through otter.ai. Participants were invited to review the transcripts so that they could make revisions or add anything that was missing. Two participants reviewed their transcripts and did not provide any suggestions for changes. TH reviewed all transcripts and developed initial codes based on concepts drawn from ecological theory, community-based health promotion and implementation science and exploratory categories that emerged from the data. KM reviewed all of the initial codes and recorded any disagreements and refinements that were needed. The two coders met to discuss coding revisions and came to consensus on the final structure and definition of higher order themes and organization of codes. All data were anonymized and stored on password-protected laptops. A summary of the results were presented to the Steering Committee members in the fall of 2021 to solicit their feedback, interpretation and recommendations for the application of findings.

## Results

This paper presents the findings regarding community engagement and adoption of the IPM. Three over-arching themes were identified within the analysis of the interview data: 1) Motivating influences, 2) Resistance to the adoption of the IPM and 3) Strategies to develop buy-in.

### Motivating influences

These findings relate to the specific rationale behind why many of the SC members became involved and what outcomes they were most interested in achieving through the implementation of the model. There was a range of motivating factors, including concerns related to substance-related harms, interest in supporting more comprehensive youth development outcomes and the potential for the development of a stronger community.

Similar to many other communities across Canada, Lanark County was experiencing the emergence of the opioid crisis and the associated harms with respect to fentanyl and other hazardous substances. This prompted community interest to explore new approaches. *“[PYLC formed] in response to an awareness that there were pockets of substance abuse, and a growing concern about the impact of fentanyl in the drug supply chain.” (SC7) The initial orientation toward an upstream prevention approach occurred through an introduction within a harm reduction strategy.* This stimulated the formation of the original group that later became PYLC.

*In February of 2017, I believe it was, there was a big meeting at one of the church auditoriums here to discuss the opioid crisis and Naloxone kits were being given out…. it was kind of a mass training. But then [Community member] and [PYLC SC member] got up and they talked maybe for about 10 minutes about this solution [the IPM]… And so that’s how it got started. (SC9).*

Community members were not only interested in the IPM because of the potential impact on substance use behaviours. They also hoped that the intervention would have more comprehensive impacts on both youth development, as well as the broader community. Some of these expectations were communicated in terms of potential wide-ranging impacts for youth and the hope that the intervention would provide support for youth who are exposed to more significant risk factors:

*I think that we would see significantly better outcomes for young people academically, socially, intellectually, in terms of scholastic achievement, in terms of reducing family conflict. (SC 8).*

Beyond the positive impacts on youth, the majority of SC members discussed the interconnections between young people, the reciprocal contributions that they can make to society and the potential for improving the health and wellbeing of the whole community. By extension, this reciprocal relationship also implicates the responsibility of the community to ensure that young people have access to important developmental opportunities.

*These are the kids who are going to be looking after us in our old age. You want somebody doped up, giving you a bath when you’re 80 years old? No, you want people who have dreams and aspirations and can have intelligent conversations and aren’t scraping by...*. *And it benefits everybody, it benefits seniors, it benefits community, it benefits everybody, because it’s the village… you know, the village is only as good as its weakest member. It’s the sad truth. And if the village won’t help its weakest members, shame on you. Shame on you. (SC9).*

### Strategies to develop buy-in

PYLC Steering Committee members (SC) described strategies they used to engage with community partners and to support buy-in with key stakeholders. There was a range of approaches that were applied by the SC members, including open communication to facilitate stakeholder engagement, leveraging existing partnerships and highlighting the value of community-based health promotion. All of these strategies served to ease concerns regarding the PYLC initiative, support relationship development and enhance integration of complementary functions across organizations and sectors.

Many of the SC members talked about the need to engage with community members by sharing information in a transparent manner while making sure to communicate any new developments and updates. They identified that it was important to do this without applying pressure for community partners to become involved or to request support at the outset.

*They went to different municipalities to let them know about what was coming or what it was all about without having any demands. So, they just went to share the information without asking specifically for money or any other resources. (SC 3).*

PYLC success was also ascribed to having strong and flexible leadership within the initiative that is attentive to partner perspectives and needs. Open communication and dialogue helped to facilitate relationships with key stakeholders.

*I think a lot of the lessons learned are that you listen to the opposition and you acknowledge and validate the opposition and ask them what they would want and you can begin to dialogue about how that can be achieved or fulfilled through this process that is people driven. So, I think that’s just people’s diplomacy skills that have been well put to the test and certainly have won a lot of friends. (SC 4).*

Many of the partnerships that were developed within PYLC had already been forged through other initiatives and this helped to facilitate the initial mobilization of the collaborative work. SC members recommended identifying overlapping goals and complementary opportunities that PYLC could provide for existing projects and other organizations. In particular, the collection of current population-based data on social determinants of health and substance use behaviours was often seen as a valuable contribution that other existing initiatives could integrate within their planning, evaluation and generation of income:

*There is lots of existing organizations and initiatives that serve the community and they’re competing for scarce resources, so when you bring a new idea to the table, it’s possible that you can be viewed as another source of competition for those scarce resources. So, I think the lesson there is that, it’s really important to identify and address those concerns as early as possible. And if there is redundancy in what’s being proposed as a new initiative, find ways to identify and, if there is a redundancy, remove it, if possible. And make it so that you are leveraging what’s going on right now, supporting what’s going on right now, and complementary to what’s going on right now… (SC 1).*

There were also several circumstances where partners came to the recognition that PYLC major objectives overlapped with their own, which created anxiety regarding whether their contribution would continue to be valued. In these situations, leadership within PYLC were careful to point out that the PYLC initiative would support the identification of community need. This was an incentive for partners who could use these findings to incentivize funders to support and sustain current intitiatives.

School boards are key partners within the IPM [18, 19]. In particular, several members described how it was important to have champions within the school board who could help to negotiate interactions and build insight with respect to the school context. Another significant approach that involved leveraging existing partnerships was to align the PYLC with the Municipal Drug Strategy (MDS). This structure facilitated the initial development of PYLC and influenced the decision to prioritize Lanark County over other regions. Part of the logic behind involving the MDS committees was to engage them in design and implementation planning when the survey data was released.

One of the key struggles for the SC members was communicating the rationale behind why a community-based health promotion approach would be beneficial, therefore, a significant part of the PYLC strategy was to build acceptance for these models. Part of this approach involves raising awareness about the benefits of ecological interventions that shift social norms, rather than individual-focused programs that recommend abstinence. We highlight factors that challenged the community engagement process, including resistance from harm-reduction/treatment advocates, and concerns regarding slow return on investments. This process involved recognizing and explaining the rationale and mechanisms of influence within community-based health promotion initiatives. SC members describe the recognition for themselves of the value of this paradigm shift from individual-focused programs to ecological system-level approaches:

*In order to address the issue of abuse of substances within that population, within that group within our within our community, we have to look at the whole community. A holistic approach is essential… So it’s not, about telling young people to stop drinking. It’s about the community and creating an environment where young people don’t feel the need to drink. To me, that’s a very significant shift in approaching the problem from the usual approach that I think we have in North America. (SC 8).*

SC members were also interested in raising this awareness with other community members in order to enhance the functioning and effectiveness of existing services. They recognized that youth substance use was a recurring issue in the community that was not being effectively addressed. This was what motivated them to become a part of the PYLC initiative:

*That’s part of why I’m so interested in getting involved with substance use initiatives so I can bring more awareness to schools because for a long time we did the whole ‘just say no’ thing or ‘this is your brain on drugs, the egg’ and we know that often really doesn’t work. (SC 3).*

Some of the process for raising awareness about the value of community-based health promotion was that there was already an existing community readiness for change that was initiated by involvement in previous innovations that emulate some aspects of the IPM approach, such as the municipal drug strategies:

*The other thing I think was fortunate, we did have municipal drug strategies in many of the communities here and so there was a willingness… to look at things in a different way and I think we had all come to the conclusion that the war on drugs doesn’t work. (SC 2).*

SC members also describe how holistic strategies, like the IPM, make intuitive sense when they are communicated to potential collaborators. They felt that part of this recognition is facilitated by having a sense of belonging and connection to community:

*There’s a conceptual framework there, that you can lay out, and that people get, right. People in the church say, ‘Well, of course, the town councillors have to be involved. And, of course, we need to have the police at the table. And of course, we need the school board to be connected, you know, it just, it makes so much sense to people because they see their community as a whole.* (SC 8).

One of the challenges the SC members faced was creating buy-in to a community-level approach that would require a long-term investment of time in order to see meaningful change.

Yet, they recognized the importance of engaging with partners who are more invested in short-term projects to integrate their contributions and energy, while managing expectations. These partners were more oriented to treatment and harm-reduction. PYLC created space for these partners as well, many of whom who bring lived experiences of substance-related harm.

*This is definitely a long-term project and some people who are quite interested in the project at the beginning, and often it’s those people who’ve personally experienced some negative impacts of problematic substance use, for example. Some of those folks are quite focussed on more short-term kinds of initiatives and what we need to do is find a way to leverage their skills, their passion and their energy. (SC 1).*

Relatedly, a key concern for PYLC was the communication of the importance of integrating prevention within an overall framework that includes harm reduction and intervention. Recognizing that partners commonly deal with significant needs and crises, it was often very difficult to shift conversations away from individual-focused interventions and to support population health initiatives that would complement the ongoing work in harm-reduction and substance use treatment.

*It was probably about 4 years ago now that I would think that word started spreading more about Planet Youth and the concept around doing prevention as opposed to looking specifically at the effects of substance use and educating more about substance use specifically. (SC 3).*

Finally, to support alignment of efforts, SC members highlighted the importance of acknowledging and communicating that prevention should not displace harm-reduction and intervention programs but balance them.

*In fact, I think we made some changes with some of them and how they are going to deal with things that maybe they’re going to focus more on prevention than harm reduction. And we made it a point of saying we don’t think one is better than the other. (SC 9).*

### Resistance to the adoption of the IPM

SC members also encountered considerable resistance to the model and we discuss the nature and influencing factors that SC members perceived to be involved in these interactions. In particular, difficulties arose related to general resistance to behaviour change, reservations about a model that was developed in another cultural context, difficulties generating buy-in to an open-ended model and stakeholder aversion to strategies that involve restriction. Some of the resistance experienced by SC members was interpreted as general unwillingness to change:

*Obviously, you’re going to have people who are resistant, right? And that’s just natural. … no one likes change. Very few people get excited about change. I mean, I think that’s part of the issue as well. (SC 2).*

*And whenever you are trying something new, people are excited for change, but the change is also hard. Some people are really against it... There is going to be resistance from some people,. (SC 3).*

SC members described experiences where other communities were reluctant to bring in an outside intervention that had not originated within Lanark County.

*Like, why are we bringing something from Iceland to Canada? You know, kind of like, why do we need to go to Iceland to, you know, to get this program and bring it back here, you know? Will it work? Iceland is a small country, how are we going to get it to work here in Lanark County? (SC 2).*

Another issue that created some difficulty for SC members in generating buy-in, was the fact that since the IPM follows a participatory approach that is developed based on the survey findings, the actual intervention functions and the specific activities to support substance use prevention were not established for Lanark County. Therefore, stakeholders questioned buying into an open-ended process that did not include recognizable strategies from the outset that could be critically assessed.

*I had a couple of municipal politicians ask me, ‘Well, you know, you’re not telling us what the solution is.’ (SC 2).*

*The challenge is in the dominant paradigm, the tendency is to look for the expert and find the answers and [the IPM] is very much bestowing upon others their expertise to voice their preferences and to help to create it. (SC 4).*

Finally, another factor that influenced community resistance was the specific tactics that were used in the IPM approach within previous implementations. Historically, the IPM has applied strategies that include restriction and environmental restructuring, such as curfews. Within interviews, SC members identified that stakeholders felt these approaches would not be feasible to implement and they would be perceived as overly restrictive.

*There was one counselor that was really against it, … she was under the impression, same as I was at the beginning, that you know, it was doing what Iceland did. Never going to have a curfew in [Community]. That’ll never work. (SC 9).*

*[SC member] is a strong advocate in control. Nothing against her, ‘These kids had too damn much time.’ So you know ‘there is a curfew on the books, we should be enforcing it.’ The cops are going ‘Wait a minute, who do you think is going to do that? It’s not us’ (SC 3).*

We also noted that these more controlling aspects of the model were particularly disagreeable to youth engagement advocates as they felt that these strategies undermine approaches that support youth empowerment and individual choice. Similar to harm-reduction advocates, these community members found it difficult to reconcile the value of interventions that are designed to manipulate the environment to support healthy behaviour change, rather than focusing on individual decision-making and capacity-building. In contrast, we have also noted a positive response to the IPM from individuals with lived experience of substance use issues. These advocates have an intimate understanding of the process and mechanisms of influence involved in the initiation of substance use as well as in the development of problematic substance use and experience of related harms. This lived experience may provide them with a strengthened ability to critically assess and recognize the potential of the IPM.

## Discussion

This paper describes some of the key factors that influenced community support for adoption of the IPM within Lanark County. These insights are critical to better understand how communities can build readiness and acceptance of the model and how to lay the foundation to support successful implementation. Research has shown that these system-wide health promotion interventions often devolve into individual-focused programming with diminished impact [1, 10], therefore it is critical to understand the specific context and factors that influence adoption and later implementation of these initiatives. Our findings capture the specific motivations that drove initial engagement with the model, the major strategies followed to support adoption and key concerns that drove resistance to the model. Below we discuss the relevance of the findings and situate them with existing research in upstream prevention and implementation science.

One of the key lessons learned that were shared by SC members was the importance of maintaining transparency and open dialogue with community members. This aligns with recommendations within implementation science research that highlight the importance of understanding community perspectives [15–17] to facilitate local buy-in [17, 46]. This approach has been demonstrated to be effective within other child and youth service collaborations [46]. In addition, researchers have identified the tension that is created when a new initiative is introduced that overlaps with the mandate of existing services [46] and there is agreement that these conflicts should be managed in a constructive way so that partners identify solutions that support ways to collaborate and leverage complementary supports [13].

Another critical issue that informs strategies to leverage existing partnerships is the consideration that partners can be in positions where they must compete for scarce resources. Research examining partnership in public health interventions have identified that lack of resources created challenges [10, 58, 59] and that this is particularly problematic for prevention efforts that are not prioritized over healthcare [59]. Similar to the PYLC steering committee members, other researchers have emphasized the importance of building on existing initiatives [58]. SC members also discussed the importance of communicating realistic timelines and managing expectations about the length of time required to achieve objectives. This challenge has been identified in other partnerships where researchers found that the time needed to build relationships exceeded the financing period [58]. Similarly, Varda [60, 61] argues that, contrary to typical practice, network potential should be measured in terms of the quality of interactions and strategic connections rather than counting the number of connections.

It is also helpful to examine the specific motivations that drove PYLC SC members to better understand which aspects of the intervention were most important to them and to explore why they chose to follow this approach. This illustrates which elements of the IPM are perceived to be of higher value and that might be most successful in generating momentum to support adoption within other contexts. Although, SC members identified a concern in the community regarding the risks related with substance use, they were also hopeful that the strategy would result in the overall healthy development of youth as well as enhanced community wellbeing.

One of the key findings is the need to argue for the inclusion of prevention strategies as an integrated component within a framework that includes treatment and harm reduction. Historically, prevention efforts have advocated for abstinence and these strategies have been identified as lacking effectiveness [2] likely not achievable in the context of widely used substances [35] and as being incongruous with youth voice [62]. In contrast, harm-reduction strategies take an inclusive approach that provides choice and are tailored to the individual [2]. As such, we encountered dogmatic views with respect to applying prevention approaches and a strong preference for harm reduction strategies.

However, the IPM takes an approach that is designed to alter risk and protective factors within youth environments, rather than applying education that solely relies on individual resistance within a social norm of substance use. The IPM represents an upstream strategy that contrasts programs that focus on individual behaviour change. Yet, advocates working in harm reduction and treatment are often dealing with crises and can be reluctant to take on a population focus. Applying the upstream analogy, that there is a fear that some are being left to drown - and these are not nameless people. Therefore, it is key to assure advocates and key stakeholders that upstream prevention approaches should not be used in isolation and that it is critical that it be combined within the framework of a comprehensive substance use strategy that applies many systems of influence [1]. This represents the combination of multiple intervention functions that support behaviour change as described by Michie [36] and the potential strengthening of impacts, as has been demonstrated in previous efforts focused on reducing tobacco use [37].

Similarly, beyond the general apprehensive reaction from stakeholders working in treatment and prevention, we have noted a hesitancy from youth engagement advocates regarding the use of curfews and other environmental restructuring strategies within the IPM. SC members identified that there was a strong negative reaction toward the curfew aspect of the previous IPM intervention tactics. Beyond feasibility considerations, strategies, such as the curfew can be perceived as undermining the ideology of youth engagement and the support of human rights. Therefore, this response is somewhat predictable, recognizing that curfews represent a restriction of human rights and freedoms. Further, shifting social norms does not follow an approach that influences intentional decision-making that aligns with youth empowerment, but rather automatic motivations (see [36]). Rappaport [63] highlighted this paradox between “rights” vs “needs” and the importance of pursuing divergent solutions that are informed by empowerment strategies. This argument is likely more relevant today with current debates regarding pandemic health restrictions and misinformation.

The IPM places a focus on risk and protective factors that influence substance use and has not yet been systematically examined to determine whether it can impact on youth wellbeing. However, previous research has demonstrated that risky behaviours are inter-related [64] and that increased exposure to risks are associated with less favourable outcomes in general [65]. In contrast, protective factors, such as a healthy family environment, supportive school climate and positive peer role models are known to reinforce each other to enhance the likelihood of positive developmental outcomes [5]. Our findings demonstrate that community partners are hopeful that the IPM will also support positive youth outcomes and wellbeing. It will be important for PYLC to examine how implementation influences wellbeing and what adaptations are needed to enhance these outcomes.

This emphasis on taking a strengths-based approach with the objectives of supporting wellbeing aligns with youth engagement and may be one of the facilitators in several communities taking the initiative to integrate youth engagement processes within their implementation approach (see [66]. It may be helpful to emphasize these impacts when seeking to generate buy-in to the model within other communities. To mitigate a lack of acceptance with youth engagement proponents, we recommend that community leaders partner with local youth, and in particular, individuals with lived experience of substance use, to integrate their perspective within decision-making processes. We have noted that individuals with lived experience of substance use issues recognize the potential of the IPM approach. Engaging young people with lived experience of key equity issues within decision-making enhances feasibility of the project, strengthens recommendations and increases impact in youth-focused initiatives [25, 67]. Further, youth advocates may be able to guide efforts that can have similar effects of reducing accessibility of substances or restriction of environments that promote risky behaviour, without the application of stronger restrictions, such as curfews. Engaging key advocates with lived experience of substance use may also help to promote acceptance among service providers working in harm reduction and treatment.

This research demonstrates that lifestyle drift is a significant risk in these communities as there are challenges with partnerships, competition for funding, opposition to moving away from individual-focused empowerment models and an urgency to consistently return to a focus on populations in need of treatment and intervention. Further, there are very few models and frameworks from the implementation science literature that can be used to support fidelity to these collaborative structures. Structures that can help support uptake involve opportunities support publication of research in this area and increased funding opportunities [1].

There is also a significant need to expand on research that applies implementation science frameworks within collaborative and complex structures to better understand how to maintain fidelity to key components within these approaches. We cannot use the same strategies that fail to change individual behaviour to try to change practice. Multiple strategies should be combined to support uptake and successful implementation of collaborative health promotion initiatives. In many circumstances, these initiatives necessitate a paradigm shift among key stakeholders to implement ecological health promotion strategies and there is very little guidance available to support collaborative initiatives. We need to develop better tools and strategies to support communities looking to adopt these initiatives.

### Limitations

We would like to acknowledge several limitations in this study. First, this data collection occurred in the early stages of the IPM process, and the PYLC Steering Committee were engaged in completing step three of 10 core steps (see [19]. As such, their insights only relate to their experiences in supporting uptake and it is not possible to predict how the initiative will develop in later stages. Yet, we suggest that it is still helpful to explore these experiences at an early stage so that members are better able to recall key events in the initial development processes.

PYLC has also been impacted by the pandemic and the related health restrictions. These are described in more detail within a related manuscript [68] and resulted in all work moving online, major delays in the planned collection of the survey and challenges with competing priorities within school and public health partners. This may influence the transferability of the findings, however, many of the reflections shared by members were based on occurrences that preceded the onset of the pandemic.

Lastly, the lead researcher was embedded within the implementation team and supports the advancement of the initiative as a collaborator and internal evaluator. Although, this method of involvement can be viewed as less objective, this practice offers several advantages including an in-depth knowledge of the context, and the ability to facilitate utilization of findings [69].

This study also demonstrates several strengths and achieves methodological trustworthiness through a variety of aspects. Taking a relativist perspective that recommends the application of flexible lists of characteristics to evaluate the value of research [70, 71], this study meets several criteria of quality, including credibility, sincerity and significant contribution (see [72]. This study is characterized by an in-depth description of context and local perspectives and is strengthened by a participatory approach (credibility). We provide a full description of the methods and reflection on the subjective lens taken (sincerity). Finally, this study stands to make several significant contributions, including a better understanding of the underlying mechanisms of influence within the IPM, support for the scaling of upstream prevention models as well as the advancement of research within substance use prevention, community-based health promotion and implementation science. This research meets each of the 21 criteria of the Standards for Reporting Qualitative Research [73].

## Conclusion

This study advances knowledge that highlights how to support uptake and scaling of the IPM as well as how to develop stakeholder buy-in to system-level innovations in health promotion and upstream prevention. This research contributes to both the fields of substance use prevention and implementation science through the description of key factors that influence the uptake of substance prevention innovations and lessons learned that can support implementation within other communities.

## Data Availability

The data that support the findings of this study are available on request from the corresponding author. The data are not publicly available due to privacy or ethical restrictions.

## Abbreviations

IPM: The Icelandic Prevention Model
PYLC: Planet Youth Lanark County
